# Exploring the Prognostic Role of D-dimer Levels in Pancreatic Cancer: A Comprehensive Review of Clinicopathological Associations

**DOI:** 10.7759/cureus.68627

**Published:** 2024-09-04

**Authors:** Sparsh Dixit, Chandrashekhar Mahakalkar, Shivani Kshirsagar, Akansha Hatewar

**Affiliations:** 1 General Surgery, Jawaharlal Nehru Medical College, Datta Meghe Institute of Higher Education and Research, Wardha, IND

**Keywords:** survival outcomes, tumor progression, coagulation, prognostic biomarker, d-dimer, pancreatic cancer

## Abstract

Pancreatic cancer is known for its dismal prognosis and high mortality rate, primarily due to late-stage diagnosis and aggressive disease progression. Finding reliable prognostic biomarkers is crucial in improving patient outcomes and guiding treatment strategies. D-dimer, a fibrin degradation product, has emerged as a potential biomarker of interest in various cancers due to its association with coagulation abnormalities. This comprehensive review investigates the prognostic role of D-dimer levels in pancreatic cancer by synthesizing current research and exploring its clinicopathological associations. Elevated D-dimer levels in pancreatic cancer patients have been linked to poorer clinical outcomes, including reduced overall survival and increased disease progression. The review examines how D-dimer levels correlate with tumor characteristics such as stage, grade, and metastatic spread, highlighting its potential utility as a prognostic marker. Additionally, the review addresses the methodological challenges in D-dimer measurement and the need for standardized protocols to enhance the reliability and applicability of results. Future research directions are identified, focusing on validating D-dimer's clinical utility and integrating it into routine practice for risk stratification and personalized treatment planning. By providing a comprehensive overview of D-dimer's prognostic value, this review aims to contribute to developing more effective management strategies for pancreatic cancer, ultimately improving patient care and outcomes.

## Introduction and background

Pancreatic cancer is a formidable challenge in oncology, marked by its high incidence and dismal prognosis [1]. As of 2024, it stands as the fourth leading cause of cancer-related deaths worldwide. Despite advancements in medical research, the five-year survival rate for pancreatic cancer patients remains critically low, hovering around 11% [2]. This poor outlook is largely due to the cancer's tendency to be asymptomatic in its early stages, resulting in diagnoses at advanced stages when treatment options are limited. The disease's aggressive nature and propensity for early metastasis further contribute to its poor prognosis [3]. The pathology of pancreatic cancer primarily involves pancreatic ductal adenocarcinoma (PDAC), a malignant tumor originating from the ductal cells of the pancreas. This cancer is driven by a complex array of genetic mutations, including those in the *KRAS*, *TP53*, *CDKN2A*, and *SMAD4 *genes. These mutations lead to unchecked tumor growth, early dissemination, and resistance to conventional therapies. Compounding these issues is the presence of a desmoplastic stroma, which not only supports tumor growth but also impedes effective drug delivery and immune system infiltration, further complicating treatment efforts [4].

In the broader context of cancer care, biomarkers play a crucial role in diagnosing, prognosticating, and managing malignancies. These measurable indicators, found in blood, bodily fluids, or tissues, provide valuable insights into disease presence, severity, and progression [5]. They can also guide therapeutic decisions and monitor treatment responses. Among various biomarkers, D-dimer has gained prominence due to its association with coagulation disorders. Elevated levels of D-dimer are commonly used to diagnose and monitor conditions such as deep vein thrombosis and pulmonary embolism. In cancer patients, increased D-dimer levels have been linked to tumor-associated hypercoagulability and poorer outcomes, suggesting its potential as a prognostic marker [6]. This review aims to investigate the prognostic role of D-dimer in pancreatic cancer by examining the existing literature and analyzing clinicopathological associations. The primary objectives are twofold: first, to explore how D-dimer levels correlate with patient outcomes, including overall survival and progression-free survival, and to assess its potential as a prognostic tool in pancreatic cancer management; second, to analyze the relationships between D-dimer levels and various clinical and pathological features of the disease, such as tumor stage, grade, and metastatic spread. By synthesizing current research findings, this review seeks to enhance the understanding of D-dimer's role in pancreatic cancer and evaluate its utility in clinical practice.

## Review

D-dimer: a biomarker overview

Definition and Biochemical Properties

D-dimer is a specific protein fragment generated during fibrinolysis, the process by which blood clots are broken down. It consists of two cross-linked D fragments of the fibrin protein, forming a dimeric structure [7]. As an essential biomarker in clinical medicine, D-dimer is crucial in evaluating thrombotic disorders such as deep vein thrombosis (DVT) and pulmonary embolism (PE). Elevated D-dimer levels in the bloodstream can indicate abnormal clotting activity, making it a valuable tool for diagnosing and managing various coagulation-related conditions [7]. D-dimer formation begins as part of the body’s response to injury. When a blood vessel is damaged, the hemostatic process is triggered to prevent excessive bleeding. This involves complex interactions among platelets, clotting factors, and the vascular system. Fibrinogen, a soluble plasma protein, is converted into fibrin strands that weave together to form a stable clot. This clot acts as a temporary barrier to control bleeding and provides a framework for tissue repair [8]. Once the injury heals, the body initiates clot dissolution, known as fibrinolysis. This process is crucial for restoring normal blood flow and preventing complications from persistent clots. Plasminogen, incorporated into the clot during its formation, is activated to plasmin, an enzyme that breaks down fibrin [9]. Various fibrin degradation products, including D-dimer, are released into the bloodstream as fibrin is degraded. The presence of D-dimer reflects the ongoing process of clot formation and breakdown, providing insight into the body’s coagulation status [9]. Measuring D-dimer levels is particularly useful in clinical settings. Elevated levels can indicate the presence of thrombotic events and a hypercoagulable state associated with various medical conditions, including cancer, infection, and inflammatory diseases [10]. However, elevated D-dimer levels are not specific to any condition; they must be interpreted with clinical findings and other diagnostic tests. D-dimer is a critical biomarker for assessing coagulation activity and guiding clinical decision-making in managing thrombotic disorders [11].

Clinical Relevance of D-dimer

D-dimer is a significant biomarker in clinical medicine, especially regarding coagulation and fibrinolysis. It is a fibrin degradation product generated during the breakdown of cross-linked fibrin, which occurs when the body dissolves blood clots [11]. The formation of a D-dimer involves several key steps in the hemostatic process. Initially, thrombin cleaves fibrinogen into fibrin monomers, which then polymerize to form a fibrin meshwork. This meshwork is further stabilized by factor XIIIa, which catalyzes the formation of covalent bonds between the D-domains of the polymerized fibrin [12]. Subsequently, plasmin breaks down the cross-linked fibrin, releasing D-dimer into the bloodstream. Elevated D-dimer levels indicate ongoing activation of the coagulation and fibrinolytic systems, reflecting a hypercoagulable state that can be associated with various pathological conditions [12]. D-dimer testing is crucial in diagnosing and monitoring thrombotic disorders in clinical practice. One of its primary applications is in evaluating venous thromboembolism (VTE), which includes conditions such as deep vein thrombosis (DVT) and pulmonary embolism (PE). In patients with symptoms suggestive of VTE, a D-dimer test can help rule out the condition, particularly in those with a low pretest probability. A negative D-dimer result has a high negative predictive value, enabling clinicians to safely exclude VTE without additional imaging studies. This streamlines patient management and reduces unnecessary healthcare costs and radiation exposure from imaging [13]. D-dimer testing is also valuable for diagnosing disseminated intravascular coagulation (DIC), a serious condition characterized by systemic activation of coagulation leading to small blood clots throughout the body. D-dimer levels can be markedly elevated in DIC due to extensive fibrin breakdown [14]. Additionally, elevated D-dimer levels can aid in diagnosing acute aortic dissection, a life-threatening condition. Beyond diagnosis, D-dimer levels can be used to monitor the effectiveness of anticoagulation therapy. A decrease in D-dimer levels in patients undergoing treatment for thrombotic events can suggest that the anticoagulation effectively resolves the clot [15]. Moreover, elevated D-dimer levels following the treatment of a thrombotic event may indicate an increased risk of recurrence. However, D-dimer results should be interpreted in the broader clinical context, as elevated levels can also occur in conditions such as pregnancy, inflammation, malignancy, trauma, liver disease, and heart disease [16]. Thus, while D-dimer testing is a valuable tool for diagnosing and managing thrombotic disorders, clinicians must consider the overall clinical picture to avoid misinterpreting results. Overall, D-dimer is a critical biomarker, enhancing the ability to effectively diagnose, monitor, and manage thrombotic disorders [17].

D-dimer in pancreatic cancer

Pathophysiology of Pancreatic Cancer and Coagulation

**Figure 1:**
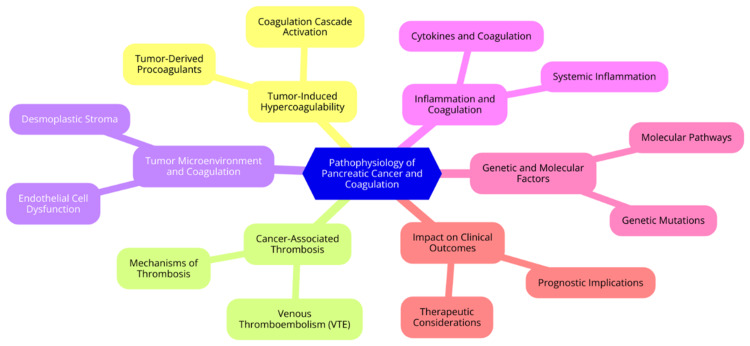
Pathophysiology of pancreatic cancer and coagulation Image Credit: Dr Sparsh Dixit

Pancreatic cancer, especially pancreatic ductal adenocarcinoma (PDAC), is strongly linked to a hypercoagulable state that can lead to thrombotic complications. The relationship between pancreatic cancer and coagulation abnormalities is complex and involves multiple pathways. A major factor contributing to this hypercoagulable state is pancreatic cancer cells' tissue factor (TF) expression [17]. TF is a potent procoagulant that initiates the extrinsic pathway of the coagulation cascade. When TF binds to factor VIIa, it forms a complex that activates factors IX and X, leading to thrombin generation and subsequent fibrin formation. This process significantly enhances the coagulation potential in patients with pancreatic cancer [18]. Moreover, pancreatic tumours release procoagulant microvesicles that promote distal thrombosis by activating the extrinsic and intrinsic coagulation pathways. These microvesicles facilitate platelet adhesion and activation, further contributing to a hypercoagulable environment [19]. Another key mechanism involves neutrophil extracellular traps (NETs), which are released upon activation of leukocytes in response to the tumour. NETs can activate the intrinsic pathway of coagulation, exacerbating the hypercoagulable state associated with pancreatic cancer. Additionally, increased plasminogen activator inhibitor-1 (PAI-1) expression in pancreatic tumours and plasma promotes hypofibrinolysis, further contributing to the hypercoagulable state [20]. Patients with pancreatic cancer often show specific coagulation changes indicative of this hypercoagulable condition. Common abnormalities include shortened activated partial thromboplastin time (aPTT), reflecting an increased tendency for clot formation, and prolonged prothrombin time (PT), indicating impaired coagulation in certain contexts. Elevated fibrinogen levels are frequently observed and are associated with tumour stage and prognosis. Increased D-dimer levels are also common in these patients, correlating with tumour stage, resectability, and overall survival outcomes [21]. These coagulation abnormalities are not only linked to thrombotic complications but also contribute to tumour progression and metastasis. The hypercoagulable state can promote angiogenesis, enhance tumour growth, and facilitate the formation of a fibrin matrix that protects cancer cells from immune system attacks. In summary, pancreatic cancer induces a complex hypercoagulable state through various mechanisms, including TF expression, tumour-derived microvesicles, NETs, and hypofibrinolysis. The resulting coagulation changes, particularly elevated D-dimer and fibrinogen levels, hold significant prognostic value in pancreatic cancer and may serve as potential biomarkers for disease monitoring and risk stratification [22]. The pathophysiology of pancreatic cancer and coagulation is illustrated in Figure 1.

Evidence of Elevated D-dimer Levels in Pancreatic Cancer

Elevated D-dimer levels have consistently been observed in patients with pancreatic cancer, particularly pancreatic ductal adenocarcinoma (PDAC). Numerous studies have shown that D-dimer levels are significantly higher in these patients compared to healthy controls and those with other types of cancer [23]. For example, a retrospective study found that the average D-dimer level in pancreatic cancer patients was 5.65 mg/L, which is markedly higher than the levels observed in breast (2.61 mg/L), gastric (2.85 mg/L), colon (4.48 mg/L), and rectal cancers (2.00 mg/L). In a cohort of 1,351 PDAC patients undergoing surgical resection, elevated preoperative plasma D-dimer levels (≥ 0.55 ng/mL) were found in approximately 30.9% of patients [24]. Another study involving 282 patients who underwent R0 resection identified that 41.5% had preoperative D-dimer levels exceeding 0.53 mg/L, highlighting the prevalence of hypercoagulability in this population [25]. The prognostic significance of elevated D-dimer levels in pancreatic cancer is considerable. Research indicates that patients with high preoperative D-dimer levels (≥ 0.55 ng/mL) experience significantly poorer overall survival (OS) outcomes. For instance, those with elevated D-dimer levels had a median OS of just 15.0 months, compared to 21.3 months for patients with normal D-dimer levels [26]. In patients who underwent R0 resection, the disparity was even more pronounced, with those exhibiting high preoperative D-dimer levels having significantly shorter OS than their counterparts. Multivariate analyses have confirmed that preoperative D-dimer is an independent predictor of OS in resectable PDAC patients, underscoring its potential utility in clinical prognostication [27]. Beyond its prognostic value, D-dimer levels also provide critical insights into tumour resectability and metastatic potential. One study identified a peripheral D-dimer cut-off of 570.6 μg/L, which showed 82.8% sensitivity for assessing the unresectability of pancreatic tumours. Moreover, metastatic PDAC patients exhibited significantly higher peripheral D-dimer levels than those with locally advanced disease (2,470 vs. 1,168 μg/L). This suggests that elevated D-dimer levels may correlate with disease progression and metastasis, making it a valuable biomarker for guiding treatment decisions [28].

Prognostic value of D-dimer in pancreatic cancer

Association with Clinical Outcomes

Elevated D-dimer levels have been consistently linked to poorer clinical outcomes in pancreatic ductal adenocarcinoma (PDAC). High preoperative D-dimer levels are significantly associated with reduced overall survival (OS) [29]. In a study involving 1,351 patients, those with elevated D-dimer levels (≥ 0.55 ng/mL) had a median OS of 15.0 months, compared to 21.3 months for patients with normal levels, marking a difference of 6.3 months (p < 0.001) [26]. Multivariate analysis further confirmed that elevated D-dimer levels independently predicted poorer OS, with a hazard ratio of 1.33 (95% CI, 1.17-1.51, p < 0.001). This indicates that D-dimer is not merely a marker of hypercoagulation but also reflects disease severity and progression [26]. Although specific studies examining the correlation between D-dimer and progression-free survival (PFS) are less common, the association between elevated D-dimer levels and poor OS suggests a likely adverse effect on PFS. Elevated D-dimer levels generally indicate a higher tumour burden and more advanced disease, which typically correlates with shorter PFS in cancer patients. Therefore, monitoring D-dimer levels may offer valuable insights into disease progression and assist in predicting patient outcomes [30]. Beyond its prognostic value, D-dimer levels may also influence treatment response and the development of resistance in PDAC. High D-dimer levels have been associated with poor responses to chemotherapy and surgical interventions. Patients with elevated D-dimer levels often present with more advanced disease at diagnosis, which complicates treatment efficacy and leads to suboptimal outcomes [29]. The hypercoagulable state reflected by elevated D-dimer levels may contribute to treatment resistance. The interaction between inflammation and coagulation pathways is crucial in cancer biology, and high D-dimer levels may indicate underlying mechanisms that promote tumour growth and resistance to therapies [30].

Relationship with Tumor Characteristics

Elevated D-dimer levels have been linked to various tumour characteristics in pancreatic cancer, including tumour stage, grade, metastatic spread, and local invasion. Studies have demonstrated a significant correlation between high D-dimer levels and advanced tumour stages in pancreatic cancer [30]. Specifically, D-dimer levels often increase with the progression of the TNM (tumor, node, and metastasis) stage, indicating that higher levels are typically found in patients with more advanced diseases. For instance, one study revealed a significant association between elevated D-dimer levels and tumour stage (P=0.0395), suggesting that as the disease advances, D-dimer levels also rise [31]. D-dimer levels are also associated with tumour grade, with higher levels observed in patients with poorly differentiated tumours. This relationship implies that D-dimer may be a marker for tumour aggressiveness, reflecting the malignancy's underlying biological behaviour [31]. Furthermore, elevated D-dimer levels are strongly associated with the presence of metastasis in pancreatic cancer. Research shows that patients with distant metastases have significantly higher D-dimer levels than those without. For example, one study reported a notable association between D-dimer levels and the extent of tumour metastasis, with statistical significance (P<0.0001) across various cancer types, including pancreatic cancer [32]. In addition, D-dimer levels correlate with local tumour invasion. Increased D-dimer levels indicate more aggressive tumour behaviour, often involving local invasion into surrounding tissues. This correlation suggests that D-dimer could potentially serve as a biomarker for assessing the invasiveness of pancreatic tumours [30]. Overall, elevated D-dimer levels in pancreatic cancer patients are significantly correlated with advanced tumour stage, poor tumour grade, and increased metastatic spread and local invasion. These associations underscore the potential of D-dimer as a valuable prognostic marker for clinical management, aiding in assessing tumour characteristics and patient outcomes. Further research is needed to elucidate these associations' mechanisms and validate D-dimer's role in clinical practice [33].

Influence of D-dimer Levels on Patient Management

Elevated D-dimer levels hold substantial implications for patient management in various clinical contexts, particularly in cancer care, including pancreatic cancer. High D-dimer levels are associated with poor prognosis in cancer patients, reflecting adverse clinicopathological features such as larger tumour size and metastasis. This correlation can guide surgical candidates' treatment decisions and risk stratification [34]. D-dimer testing aids healthcare providers in assessing the likelihood of thromboembolic events, such as deep vein thrombosis (DVT) and pulmonary embolism (PE). In patients with elevated D-dimer levels, further diagnostic imaging may be necessary to rule out these conditions, potentially influencing treatment plans, including the need for anticoagulation therapy. Combining D-dimer levels with other markers, such as CA19-9 and the neutrophil-lymphocyte ratio (NLR), can enhance prognostic accuracy and enable more tailored treatment strategies based on individual risk profiles [7]. Given its prognostic value, there is a strong case for routinely including D-dimer testing in the preoperative assessment of patients with pancreatic cancer. Its ability to predict outcomes and inform treatment strategies could improve patient management and survival rates. D-dimer testing can be integrated into multi-test algorithms to evaluate patients with suspected thromboembolic complications, streamline decision-making in emergency settings and oncology practices, and ensure timely and appropriate interventions [35]. However, D-dimer’s specificity is limited, as elevated levels can occur in various conditions beyond thromboembolic disease, including infections and inflammation. Therefore, interpreting D-dimer results requires clinical judgment and correlation with other diagnostic findings. In summary, incorporating D-dimer testing into clinical practice offers significant potential for enhancing patient management in pancreatic cancer and other malignancies. Its role in risk stratification and treatment planning can lead to more personalized care and improved outcomes. Continued research and the development of standardized protocols will be essential to maximizing the utility of D-dimer in oncology [36].

Clinicopathological associations

Demographic and Clinical Factors

A range of demographic and clinical factors, including age, gender, and comorbidities, influence D-dimer levels. Understanding these influences is crucial, particularly in the context of pancreatic cancer and related health conditions [36]. Research shows that D-dimer levels tend to increase with age. In elderly populations, particularly those over 65, elevated D-dimer levels are commonly observed and are associated with higher rates of thrombotic events and mortality. This rise in D-dimer levels among older adults is believed to be linked to age-related changes in the coagulation system, including increased thrombin generation and fibrin formation. Additionally, functional status plays a role; individuals with greater functional impairment often exhibit elevated D-dimer concentrations, reflecting underlying health issues related to ageing [37]. While studies have shown that D-dimer levels can vary between genders, these differences are often not statistically significant. For instance, median D-dimer values are generally similar between men and women, although some research suggests that men may have slightly higher levels. Despite these similarities in D-dimer concentrations, men may experience worse clinical outcomes in certain conditions, such as COVID-19. This observation implies that factors beyond D-dimer levels, including biological differences and social determinants of health, contribute to disparities in clinical outcomes [38]. Comorbidities such as diabetes and cardiovascular diseases significantly affect D-dimer levels. Elevated D-dimer levels are frequently observed in patients with diabetes, with studies indicating that diabetic individuals often have higher median D-dimer values compared to non-diabetics. The relationship between diabetes and increased D-dimer levels may be due to the inflammatory state associated with the disease, which can enhance coagulation [39]. Similarly, conditions like hypertension are predictive of elevated D-dimer levels. Patients with a history of cardiovascular disease often show increased D-dimer concentrations, indicating a heightened risk of thrombotic events. Multiple comorbidities generally correlate with higher D-dimer levels, suggesting that an individual’s overall health status plays a crucial role in D-dimer elevation [40].

Laboratory and Imaging Correlations

The relationship between D-dimer levels and imaging findings, along with their interaction with other laboratory markers, plays a crucial role in understanding the clinical implications of conditions such as pancreatic cancer and pulmonary embolism. Elevated D-dimer levels have been linked to increased severity in imaging findings, particularly in computed tomography pulmonary angiography (CTPA). For example, a study found that D-dimer levels positively correlated with the pulmonary artery obstruction index (PAOI), suggesting that higher D-dimer levels reflect a greater severity of pulmonary embolism (PE) as seen on imaging [41]. In patients with acute conditions like SARS-CoV-2 pneumonia, higher D-dimer levels have been associated with elevated chest CT severity scores. Patients with D-dimer levels exceeding a certain threshold demonstrated significantly higher CT scores and more extensive lung involvement, indicating that D-dimer can serve as a biomarker for assessing the severity of lung pathology. Conversely, some studies suggest that D-dimer levels may not strongly correlate with certain imaging parameters. For instance, a study on pulmonary embolism revealed weak correlations between D-dimer levels and the diameter of the pulmonary trunk, indicating that while there may be some association, it is not robust enough to be clinically predictive [42]. D-dimer levels also interact with other laboratory markers, enhancing prognostic capabilities. In pancreatic ductal adenocarcinoma, combining preoperative D-dimer and fibrinogen levels has been shown to offer a more comprehensive prognostic indicator for patient outcomes following surgical resection. Elevated D-dimer levels in cancer patients can reflect a hypercoagulable state, which is often compounded by other inflammatory markers. This interplay can complicate the interpretation of D-dimer results, as elevated levels may be due to a range of underlying conditions, including malignancy, infection, or thrombosis [43]. Integrating D-dimer levels with other laboratory tests can aid risk stratification and treatment planning. For instance, D-dimer testing can help determine the need for further imaging studies like CTPA in patients suspected of having venous thromboembolism. However, its specificity may sometimes lead to overutilization. The variability in correlation strength across different studies highlights the importance of carefully interpreting D-dimer results in conjunction with other clinical data [44].

Methodological considerations

Variability in D-Dimer Measurement

Variability in D-dimer measurement presents significant challenges in clinical practice, especially concerning pancreatic cancer prognosis. This variability stems from differences in assay methodologies and issues related to standardization, which can affect the consistency and comparability of results across studies [26]. A major source of variability is the range of assay methods used to measure D-dimer levels. Different assays, such as microlatex-enhanced assays, enzyme-linked immunosorbent assays (ELISA), and other immunoassays, utilize varying monoclonal antibodies and detection systems. This diversity can result in substantial discrepancies in reported D-dimer levels. For example, studies have demonstrated that results can vary significantly when the same samples are tested using different platforms. This highlights the challenge of relying on D-dimer as a universal biomarker for clinical decisions. These discrepancies can complicate the interpretation of D-dimer levels regarding patient prognosis and treatment planning [13]. Standardization of D-dimer assays is crucial to ensure that results are comparable across laboratories and clinical settings. Several studies have sought to address this issue by developing harmonization protocols. One approach involved calculating conversion factors for different assays to improve correlations among results and reduce inter-assay variability. However, despite these efforts, significant variability persists due to the complex nature of D-dimer as a degradation product of cross-linked fibrin. This complexity includes a wide range of molecular forms, making it difficult to establish a uniform standard for measurement [45]. The implications of this variability are extensive. In clinical decision-making, inconsistent D-dimer levels can lead to misinterpretation of a patient's coagulation status, potentially impacting treatment decisions in critical scenarios such as cancer surgery or the management of thromboembolic events. In research, differences in assay methodologies can impede the comparability of findings across studies. For instance, when assessing the prognostic value of D-dimer in pancreatic cancer, results may seem contradictory if different assays produce disparate D-dimer levels for the same patient group. This inconsistency can create confusion in the literature and complicate the development of clinical guidelines [46]. Ultimately, addressing the variability in D-dimer measurement through standardization and harmonization of assay methods is essential for enhancing the reliability of D-dimer as a prognostic marker in pancreatic cancer. Future research should focus on developing universally accepted protocols and guidelines to improve the clinical utility of D-dimer testing. Such advancements will ensure that D-dimer levels provide meaningful insights into patient prognosis and treatment strategies, ultimately improving outcomes for individuals with pancreatic cancer [26].

Study design and limitations

In studying the prognostic role of D-dimer levels in pancreatic cancer, it is crucial to understand the various study designs and their inherent limitations to accurately assess the reliability of findings. Cohort studies are observational and track individuals over time to evaluate the incidence of outcomes based on their exposure status [47]. These studies can be prospective, following subjects from the point of exposure forward, or retrospective, where past exposure data is analyzed to assess current outcomes. Cohort studies excel in establishing temporal relationships between exposure and outcome and are well-suited for investigating multiple outcomes from a single exposure. However, they often require large sample sizes and lengthy follow-up periods, making them both costly and time-consuming. Additionally, loss to follow-up can introduce bias, potentially affecting the study’s internal validity [47]. Case-control studies start by identifying subjects based on their outcome status (cases with the disease and controls without) and then looking back to evaluate exposure to potential risk factors. These studies are particularly efficient for examining rare diseases or those with long latency periods and are generally quicker and less expensive than cohort studies. Nevertheless, their retrospective nature makes them prone to biases, such as recall bias, where participants may inaccurately remember past exposures. They also cannot definitively establish causality due to their backward-looking design [48]. Nested case-control and case-cohort studies are variations of traditional cohort and case-control studies. In a nested case-control study, controls are selected from cohort participants who had not developed the outcome when cases were identified. A case-cohort study involves selecting a random subcohort from the main cohort, allowing for examining multiple outcomes. These designs are more efficient and cost-effective than traditional cohort studies and can study multiple outcomes using the same controls. However, they may be susceptible to selection bias if the controls are not representative of the general population, and the complexity of the design can complicate interpretation and analysis [49]. Common limitations and biases in current research include selection bias, where the study participants are not representative of the general population; recall bias, particularly prevalent in case-control studies, where participants may inaccurately recall past exposures, leading to differential reporting between cases and controls; loss to follow-up in cohort studies, which can affect the study’s conclusions if a significant number of participants drop out; confounding variables, which can distort the observed relationship between exposure and outcome; and misclassification bias, which occurs when participants are incorrectly categorized regarding their exposure status or outcome, potentially diluting the true association between the variables being studied [50].

Future directions

Emerging research and innovations in D-dimer measurement and technology promise to enhance the clinical utility of this biomarker. Recent advancements in biosensing technology have led to the development of rapid D-dimer testing methods. For example, a study proposed using giant magnetoresistance sensors combined with microfluidics to improve the sensitivity and speed of D-dimer tests, potentially enabling point-of-care applications. Additionally, innovations in electrochemical sensors, such as those incorporating molecularly imprinted polymers, are being explored to enhance the specificity and accuracy of D-dimer assays. These technologies aim to reduce inter-laboratory variability and improve the reliability of results across various clinical settings [51]. Emerging evidence suggests that D-dimer may also be useful in diagnosing conditions beyond traditional thromboembolic disorders, such as acute aortic dissection and cerebral venous thrombosis, thereby expanding its clinical utility. The integration of D-dimer testing in managing COVID-19 has garnered attention, as elevated levels correlate with disease severity and can inform treatment strategies. This underscores the need for further research into D-dimer's role across various clinical contexts, particularly in oncology and infectious diseases [52]. Current guidelines from organizations like the American Society of Hematology and the European Society of Cardiology support using D-dimer testing for patients with low to intermediate pretest probability of venous thromboembolism (VTE). These guidelines emphasize the importance of employing highly sensitive assays to maximize negative predictive value. Standardizing D-dimer assays is crucial, as variability in assay performance can lead to diagnostic errors, especially in cancer patients where D-dimer levels may be affected by both malignancy and treatment regimens. Establishing uniform cut-off values and pretest probability models tailored to specific patient populations is essential for improving clinical outcomes [53]. There is an urgent need for multicenter trials to validate the effectiveness of novel D-dimer assays and explore their prognostic capabilities in diverse patient populations, particularly in cancer and COVID-19 contexts. Research should also focus on harmonizing D-dimer measurement techniques to establish consensus on reporting standards and clinical interpretation, enhancing integration into routine clinical practice and improving patient care [54].

## Conclusions

In conclusion, the prognostic role of D-dimer in pancreatic cancer offers promising insights into enhancing patient management and outcomes. Elevated D-dimer levels have emerged as a significant indicator of tumor-related hypercoagulability and may correlate with adverse clinical outcomes, including reduced survival rates and disease progression. The association between D-dimer levels and various clinicopathological features, such as tumor stage and metastatic spread, underscores its potential utility as a prognostic marker in pancreatic cancer. As research continues to evolve, integrating D-dimer testing into routine clinical practice could provide valuable prognostic information, aiding in risk stratification and personalized treatment planning. However, further studies are needed to standardize measurement techniques, validate clinical utility, and explore the full potential of D-dimer as a tool for improving patient outcomes in pancreatic cancer. Ultimately, a better understanding of D-dimer’s role could contribute to more effective and individualized approaches to managing this challenging disease.
